# Higher and Lower Pleasures Revisited: Evidence from Neuroscience

**DOI:** 10.1007/s12152-017-9339-2

**Published:** 2017-07-08

**Authors:** Roger Crisp, Morten Kringelbach

**Affiliations:** 1 St Anne’s College, Oxford, UK; 2 The Queen’s College, Oxford, UK

**Keywords:** Pleasure, J.S. Mill, Neuroscience, Well-being, Hedonism, Happiness

## Abstract

This paper discusses J.S. Mill’s distinction between higher and lower pleasures, and suggests that recent neuroscientific evidence counts against it.

In Plato’s *Republic* ([1]: 580d-588a), Socrates suggests that there is a particular kind of pleasure peculiar to each part of the human soul, taken respectively in profit, honour, and knowledge and its acquisition. When deciding on their relative value, Socrates suggests, we should listen only to the person whose soul is governed by reason, since the philosopher’s position is based on experience of the various types of pleasure, on reason, and on argument. The philosopher’s view is that ‘lower’ pleasure is far less valuable than the higher, and that we should pursue it only in so far as it is necessary for survival. Socrates goes on to suggest that most ‘lower pleasure’ isn’t really pleasure at all: it is merely absence of pain, which looks positively pleasurable only because of the contrast with pain. He calculates that the life of a philosopher king will be 729 times as pleasant as that of a tyrant (the life governed by desire for the lowest pleasures). The mathematics here is hard to understand, but Socrates’ position is a clear example of what we shall call the *higher/lower thesis*, according to which one kind of pleasure (in this case, intellectual pleasure) is more valuable than another (bodily pleasure).

Socrates uses the higher/lower thesis to argue that the life of the philosopher – which is good because of its intellectual aspects – is also the most pleasant. John Stuart Mill, almost certainly with this passage of the *Republic* in mind, puts both theses to a quite different use: to defend hedonism. In chapter 2 of *Utilitarianism*, having outlined utilitarianism itself, Mill goes on to discuss various objections to the doctrine. The first is to its hedonism: that, in postulating no end other than pleasure, hedonism is ‘utterly mean and grovelling … a doctrine worthy only of swine’ ([2]: 2.3). Rather, we are meant to assume, there are non-hedonistic goods available to human beings, and these are ‘higher’ than pleasure.

The standard Epicurean response to this charge, Mill claims, is to draw a distinction between the pleasures available only to human beings and those available also to non-humans (2.4):

Human beings have faculties more elevated than the animal appetites, and when once made conscious of them, do not regard anything as happiness which does not include their gratification… [T]here is no known Epicurean theory of life which does not assign to the pleasures of the intellect, of the feelings and imagination, and of the moral sentiments, a much higher value as pleasures than to those of mere sensation.

Mill claims that most utilitarian writers have ranked mental over bodily pleasures primarily on the basis of certain accidental features, such as permanence or cost. He does not object to such a ranking but insists that utilitarians might also have taken the ‘higher ground’:

It is quite compatible with the principle of utility to recognise the fact, that some *kinds* of pleasure are more desirable and more valuable than others. It would be absurd that while, in estimating all other things, quality is considered as well as quantity, the estimation of pleasures should be supposed to depend on quantity alone.

It is important to note that by ‘quantity’ here, Mill does not mean ‘quantity of pleasantness’. The end of paragraph 2.8 – in which he contrasts quality with quantity as understood in terms of intensity -- makes it clear that he has in mind *intensity*; and again intensity here is to be equated not with *degree* of pleasantness, but with the *intensity* of the pleasurable sensation itself. The degree of pleasantness will rise with intensity, but will be determined by other factors – not least, duration: a longer enjoyable experience, other things equal, is more pleasant, and hence more choiceworthy, than a shorter. But factors other than duration may also be relevant, and this brings in the notion of quality. It is almost certain that Mill would have included the pleasures of classical music among the higher pleasures: they are available only to humans, and involve the feelings and the imagination, as well as the intellect. Consider, for example, the ‘extreme pleasure’ Mill ‘drew from the delicious melodies’ of Weber’s Oberon, when recovering from his depressive illness of 1827–8 ([3]: 122). This pleasure is higher than that of, say, lemonade on a hot day because of its nature, and its being higher results in its being more pleasant, despite any greater intensity that might be found in the experience of lemonade.

Mill resurrects Socrates’s epistemological argument, claiming that whether or not a pleasure is higher is a matter for ‘competent judges’ (2.5–6, 8). But he goes beyond Socrates in introducing a discontinuity into the assessment of higher pleasures:

If one of the two [pleasures] is, by those who are competently acquainted with both, placed so far above the other that they prefer it, even though knowing it to be attended with a greater amount of discontent, and would not resign it for any quantity of the other pleasure which their nature is capable of, we are justified in ascribing to the preferred enjoyment a superiority in quality, so far outweighing quantity as to render it, in comparison, of small account.

The details of Mill’s view are not entirely clear. But the general gist seems to be that human beings, who experience pleasures of the intellect and so on, would not give these pleasures up for ‘the fullest allowance of a beast’s pleasures’.

Mill’s argument is meant, perhaps, not as a direct response to the anti-Epicurean objection, but as an attempt to defuse it. The anti-Epicureans appear to believe that the life recommended by Epicureans is one of animal or bodily pleasure, whereas in fact Epicureans will recommend just the kind of life the objectors find most valuable, in which intellectual and other forms of higher pleasure are fully, indeed maximally, represented. There is of course more work to be done, since, as we understand the anti-Epicureans, they are claiming not merely that pleasure is bestial, but that intellectual activities and so on are valuable independently of pleasure. That work Mill does later in *Utilitarianism*, in the third stage of his notorious ‘proof’ of utilitarianism, according to which we desire nothing other than pleasure (4.4–11). But even at this point in the argument he might hope that at least some of those who have accepted the anti-Epicurean conclusion will give it up once they recognize that hedonists can place superior human pleasures in a category quite separate from bodily pleasures.

At this point, we need to consider how pleasure is understood from the philosophical perspective. On the most straightforward understanding, pleasure is a *feeling*. It has been common in recent years for philosophers to distinguish between *internalist* accounts of pleasure, according to which pleasure is an introspectively discernible sensation common to all pleasurable experiences, and *externalist* accounts, according to which there is no such pleasurable ‘feeling tone’ but pleasures consist in experiences towards which the subjects have a certain pro-attitude (such as that the experience continue) (see [4]: 87–91). Consider, say, the pleasure of listening to Oberon. Internalists will claim that the listener experiences a certain sensation, that of pleasure; externalists will deny that, claiming that she experiences the sound of the music, and takes a positive attitude toward it.

This example brings out, we suggest, the main problem with externalism. It fails properly to capture the idea that pleasure is a feeling. The most that can be salvaged from such accounts is the idea that pleasure is the feeling of having an experience towards which the subject has the relevant pro-attitude. But even this relic of externalism should be rejected, since it fails to explain the common case in which someone justifies the attitude in question with reference to the pleasurableness of the experience.^1^

Support for externalism is often sought in the fact that pleasures – that is, pleasurable experiences – differ greatly in kind from another: compare appreciating Oberon, drinking lemonade on a hot day, and playing basketball. But internalists will of course not deny this. The fact that experiences differ in kind fails to show that their being pleasurable does not consist in a single felt quality. People’s comparative judgements seem to bear this out. Having spent a few hours engaged in the three activities just mentioned, I might well rank the Oberon as more enjoyable than the lemonade, and the lemonade as more enjoyable than the basketball. I might say: ‘I got more pleasure from the Oberon than from either of the other two activities. The Oberon, that is, was the most pleasurable’. The error of externalists who rely on the great variation that is found in pleasurable experiences could be seen as a failure to distinguish between *pleasures* and *pleasure* (see [5]). My pleasures differ greatly; one important thing they have in common is that they all involve pleasure. And it is their all involving pleasure that explains why we call them pleasures.

Internalism, understood in its most straightforward form, seems on the face of it to be in some tension with the higher/lower thesis. In so far as pleasures are valuable *as pleasures*, it seems that they all involve the same sensation, pleasure. But perhaps things are more complicated. There may be, perhaps, two kinds of sensation in play -- higher pleasantness, and lower pleasantness – and the mistake of those who deny the higher/lower thesis consists in failing to see the distinction between the two and perhaps also how one kind of sensation cannot, from the evaluative point of view, simply be traded against the other.

Here it might be worth shifting the debate into another discipline. If there are two such sensations, we might expect them, purely on the basis of supervenience considerations, to differ significantly in their underlying physiological features. If we found that the brain states underlying the *pleasantness* of higher pleasures were radically different from those underlying that of lower pleasures, this would be major piece of evidence in favour of the higher/lower thesis, or at least in favour of the view that denying that thesis based on internalism would too hasty.

What, then, do we know at this point about the neuroscience of pleasure? Consider two possible views of pleasure and the brain. The first we might call the *bolt-on view*, according to which experiences become pleasurable through the activation of a certain pleasure circuit, or certain circuits, common to all pleasurable experiences. Listening to music, drinking, and playing sport become pleasurable simply through the addition, or the bolting on, of the activation of the pleasure circuit to the original experience. On the *dissimilarity view*, however, the bolt-on position is false. There is no single pleasure circuit, or set of pleasure circuits, common to all pleasures. The proponent of the bolt-on hypothesis might claim, for example, that the sensation of pleasure evolved first in connection with certain activities that had high survival value, such as those of eating and sex, but then later became correlated with other experiences, such as those of music or playing games. According to the dissimilarity view, this has not happened, since the underlying physiology of pleasantness differs between different pleasures.

It should be obvious that the bolt-on view makes the higher/lower thesis much harder for a hedonist like Mill to maintain. Much of the neuroscientific evidence is of course not yet in,^2^ but at present the tenor of research suggests that the neural substrate of pleasure in quite different kinds of activity is quite similar. According to Kent Berridge and Morten Kringelbach ([8]: 649; see also [9]: 4; [10]: 2):

The experience of one pleasure often seems very different from another. Eating delicious foods, experiencing romantic or sexual pleasures, using addictive drugs, listening to music, or seeing a loved one: each feels unique. The only psychological feature in common would seem that all are pleasant. However, the difference in one’s subjective experiences is not necessarily a good guide to the underlying neural mechanisms. Those neural mechanisms may overlap to a surprising degree.

Over the last decades, a growing set of results from neuro-imaging studies have suggested that many diverse rewards activate a shared or overlapping brain system: a ‘common currency’ reward network of interacting brain regions. Pleasures of food, sex, addictive drugs, friends and loved ones, music, art, and even sustained states of happiness can produce strikingly similar patterns of brain activity. These shared reward networks include anatomical regions of prefrontal cortex, including portions of orbitofrontal, insula, and anterior cingulate cortices, as well as often subcortical limbic structures such as NAc, ventral pallidum (VP), and amygdala …. An implication of the common currency hypothesis is that insights into brain hedonic substrates gained by experiments using one kind of pleasure, such as food ‘liking’, may apply to many other pleasures too.

No significant disagreement with these claims is represented in recent neuroscientific literature, and last year Berridge received an award for Distinguished Scientific Contribution from the American Psychological Association. In other words, current neuroscience seems to favour the bolt-on thesis, and hence the denial, for the present at least, of the higher/lower thesis. Note also three further points.

First, pleasure-responses in many different kinds of activity, including intellectual and bodily activities, often overlap, the most obvious response, of course, being the smile. This suggests that they are at least using the same output pathways, and that we might reasonably expect the system generating the pleasure to be the same in each case.

Second, and more significantly, evolutionary theory suggests that we should *expect* there to be substantial neural overlap between the circuits underlying both kinds of pleasure. Evolution is a tinkerer, and parsimonious in so far as it would not ‘waste’ resources on developing new circuitry when it could build on that which already existed. To date, no evidence has been found of divergent networks for processing a particular kind of pleasure, such as intellectual pleasure.

Third, and relatedly, consider the commensuration of different kinds of pleasure. From a computational point of view, rather than having separate circuits, it would be far more efficient to have a common currency of pleasure, which could be used for the comparisons needed for decision-making. In order effectively to allocate brain resources it is important for the brain at all times to try to optimize the energy spent on pursuing things allowing survival as individuals and as a species (Kringelbach, Green, and Aziz [11]). We need to be able to decide when we can safely listen to Oberon or play basketball at the expense of looking after our progeny or staving off starvation. It might be objected that when making decisions we do not standardly compare *actual* pleasure or pleasures, that is, currently experienced pleasure or current pleasurable experiences, but *representations*. This claim is, of course, true, but it remains the case that these representations are of pleasurable experiences, and that comparing two representations may be simpler if they are representations of the same thing. Other things equal, if you ask me to rank in quality two pieces of fruit, my choice will be easier if you show me two pictures of two different apples than if you show me a picture of an apple and a picture of an orange.

In other words, the most plausible hypothesis is that, though higher pleasures of course involve higher-level brain activity, especially cognitive activity, there is no good reason for thinking that the *pleasure* of higher pleasures does not correlate with the same neural networks as lower, sensory pleasures. But it may now be argued that the *combination* of activating the pleasure-circuits *and* activating certain cognitive processes leads to an entirely different kind of *experience*, which is in a sense more than the sum of its parts. An intellectual or higher pleasure is still, perhaps, the ‘bolting on’ of the activation of some independent kind of pleasure-circuit – as found in sensory pleasures – to higher-level, cognitive experiences, but its *subjective* nature has been transformed through cognition. Further, one might claim that phenomenal consciousness is a feature of the whole brain (see [12]: 229). And of course it will be denied by no one that the neural substrate of intellectual pleasures differs from that of bodily pleasures, in so far as intellectual and bodily experiences correlate with different parts of the brain. So it may well be that human conscious *experience* of pleasure is different not only in degree but also in kind from that of other animals, primarily because of the advanced cognitive abilities of the human brain.

At this point, it should be noted first that humans are not particularly good at introspection. We have the ability to fool both others *and* ourselves about our subjective experiences and motives. Nevertheless, activities combining sensory and social pleasures such as those involved in a dinner party could have a synergistic effect on the higher-order pleasures experienced in humans, which might be hard to find in other animals. But until we understand a lot more about the effects on phenomenal consciousness of the co-instantiation of different kinds of brain activity (such as cognition and the activation of the pleasure-circuits), this question must remain largely one for introspection and a priori argument.

In our experience, the kind of *pleasure* we take in, say, drinking lemonade is pretty much exactly the same as that we take in listening to music. Those experiences *as a whole* are very different from one another, of course, but not in so far as they are pleasurable. Both experiences feel good or ‘positive’ – and that feeling good, if we understand ‘feeling’ in a broad sense, just is what pleasure amounts to.^3^ And this, as we have suggested above, explains why it is sometimes so easy to compare two very different kinds of pleasurable experience.

Further, the parsimony-related arguments above apply also at the level of the whole brain. If there *is* some phenomenal difference between intellectual and bodily pleasures which cannot be put down to the different neural correlates of cognition and sensory experience, that difference must correlate with certain physically realized relations between cognition and the pleasure-circuits on the one hand, or sensory experience and the pleasure-circuits on the other hand, that do not consist merely in co-existence. And these physical substrates would themselves have to be of different kinds, to explain the difference at the phenomenal level. But we would expect evolution not to produce such physical substrates unless they are necessary. And as far as we can see, there is no reason to think that mere co-existence (as described by the bolt-on view) would not be functionally sufficient. Further, important phenomenal differences, if they correlate with the lack of any common neural currency, might be expected to make comparisons of pleasures more difficult than they appear to be, and themselves more costly in terms of expenditure of evolutionary energy.

In summary, then, neuroscientific evidence for the higher/lower thesis is currently lacking. This does not mean that it may not emerge when and if new brain imaging technologies and methodologies of interpreting images emerge. But what is clear is that the existing data show that: 1) intellectual pleasure and bodily pleasure share final common pathways in the ensuing reactions to pleasurable stimuli irrespective of type; 2) evolution appears to have conserved and re-used the neural pleasure mechanisms used for bodily pleasures when dealing with intellectual, social, and other more complex pleasures; and 3) the existence of a common currency allows for pleasures of different kinds to be compared and acted upon.^4^

## Footnotes

1 For further discussion, see [6]: 103–11.

2 For a good survey, see [7].

3 We are not arguing that pleasure is some kind of very specific sensation, such as an itch. Rather we seeing it as equivalent to enjoyment, which we believe any plausible view must see as an aspect of felt experience rather than as a matter of mere attitudes or behaviour.

4 For comments and/or discussion, we are grateful to participants at a conference on hedonism convened by Bastian Stern at Ertegun House, Oxford, in March 2014, Ian Phillips, and two anonymous reviewers for this journal.
